# Supplementary material for: Effect of Short-Chain Fatty Acids and Polyunsaturated Fatty Acids on Metabolites in H460 Lung Cancer Cells
Source: Molecules. 2023 Mar 3;28(5):2357. doi: 10.3390/molecules28052357 (PMC10005177; doi:10.3390/molecules28052357)
Supplement: Supplementary file 1 [file molecules-28-02357-s001.zip › molecules-2171777-supplementary.pdf]

Supplementary Materials

Effect of Short-Chain Fatty Acids and Polyunsaturated Fatty Acids on Metabolites in H460 Lung Cancer Cells

Tianxiao Zhou, Kaige Yang, Jin Huang, Wenchang Fu, Chao Yan * and Yan Wang *

School of Pharmacy, Shanghai Jiao Tong University, Shanghai 200240, China
* Correspondence: chaoyan@sjtu.edu.cn (C.Y.); wangyan11@sjtu.edu.cn (Y.W.); Tel.: +86-21-3420-5673 (C.Y.); +86-21-3420-5673 (Y.W.)

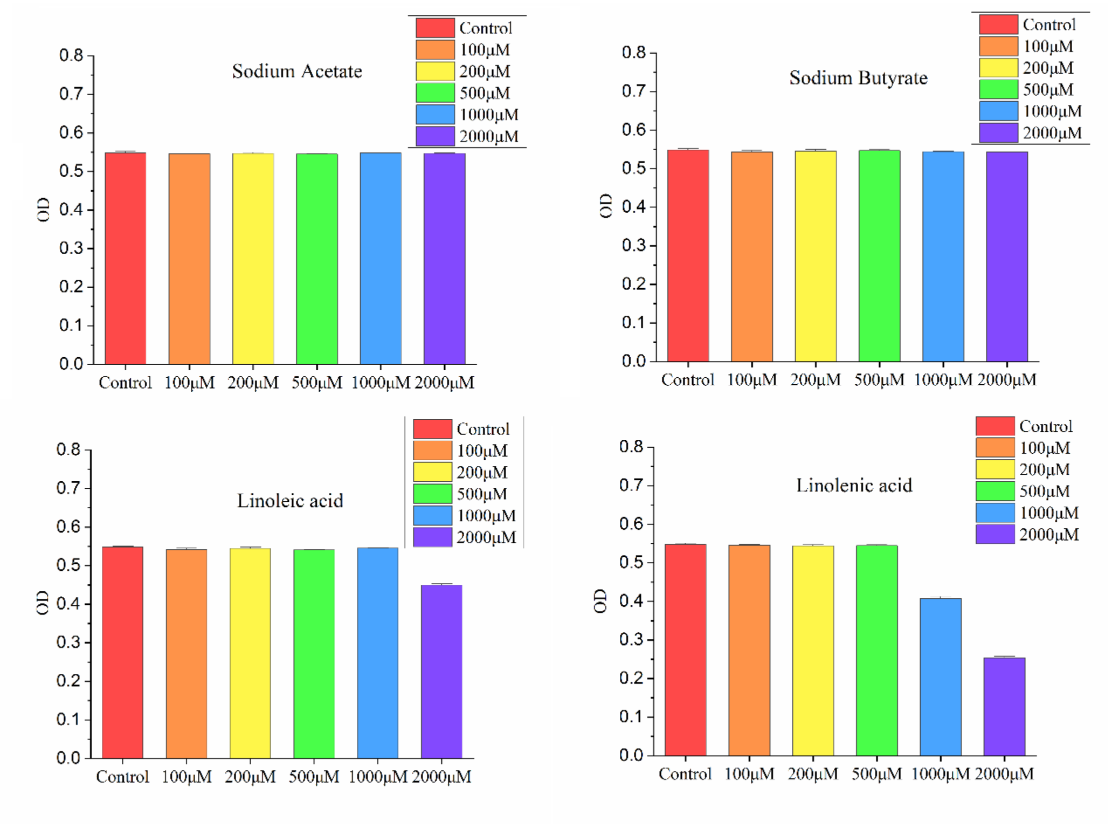

Figure S1. CCK8 experimental data of four fatty acid groups.

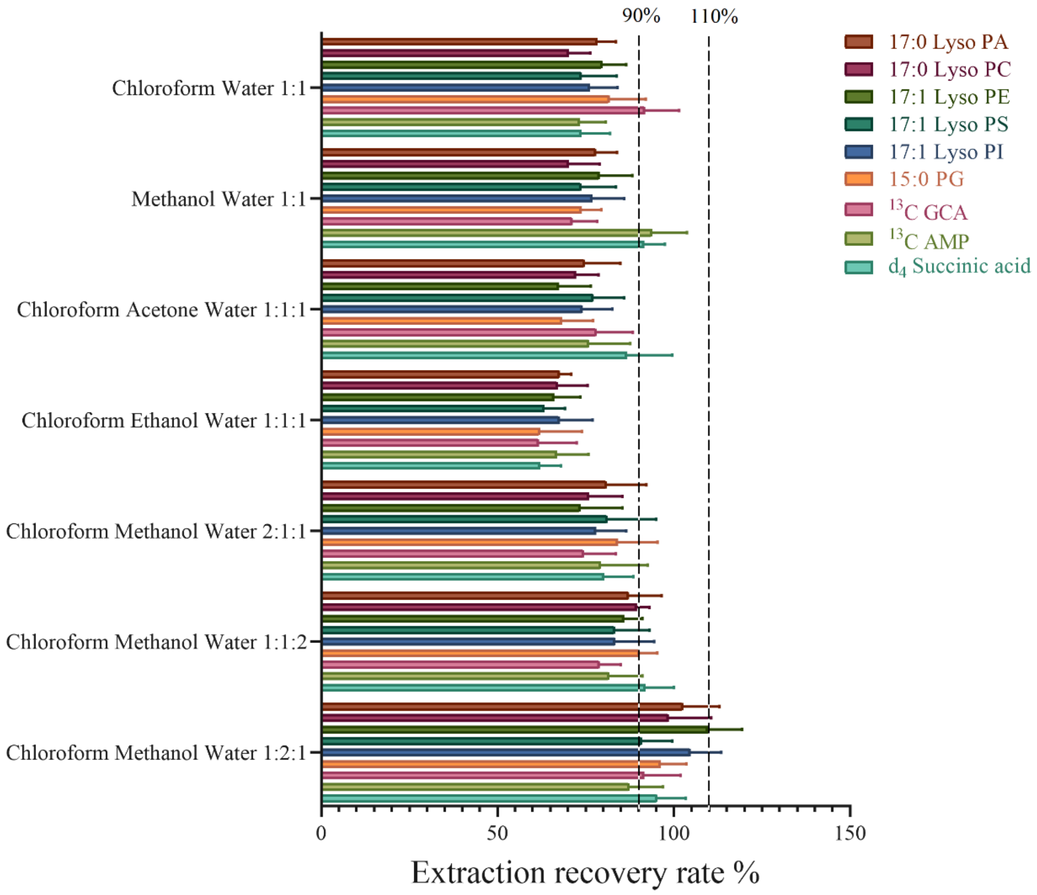

Figure S2. The extraction efficiency of different extraction solvent systems in H460 lung cancer cell.

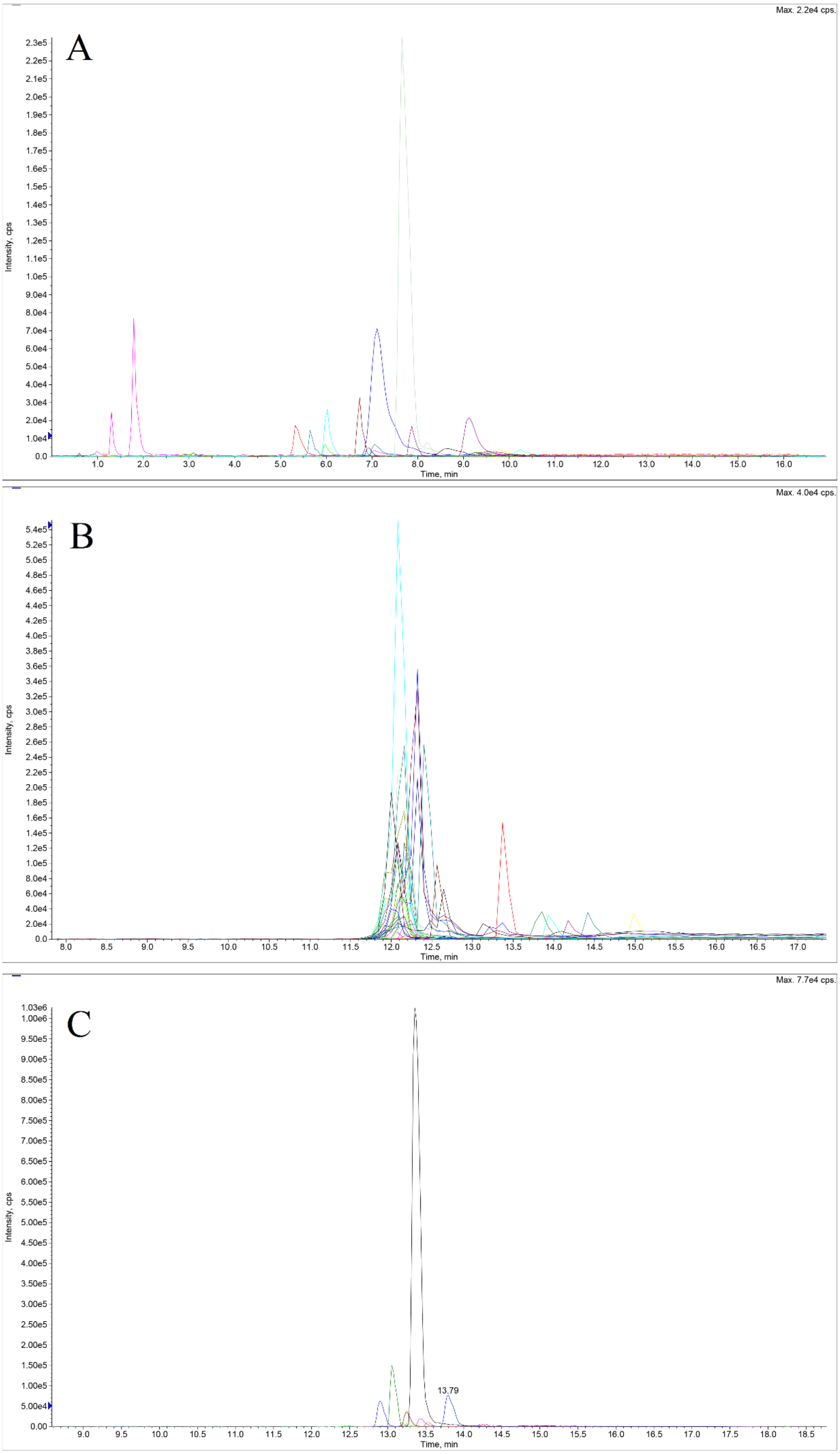

Figure S3. Chromatograms of mix-standards: (A) energy metabolites, (B) Phospholipids and Bile acids, ESI+; C: Phospholipids and Bile acids, ESI-.

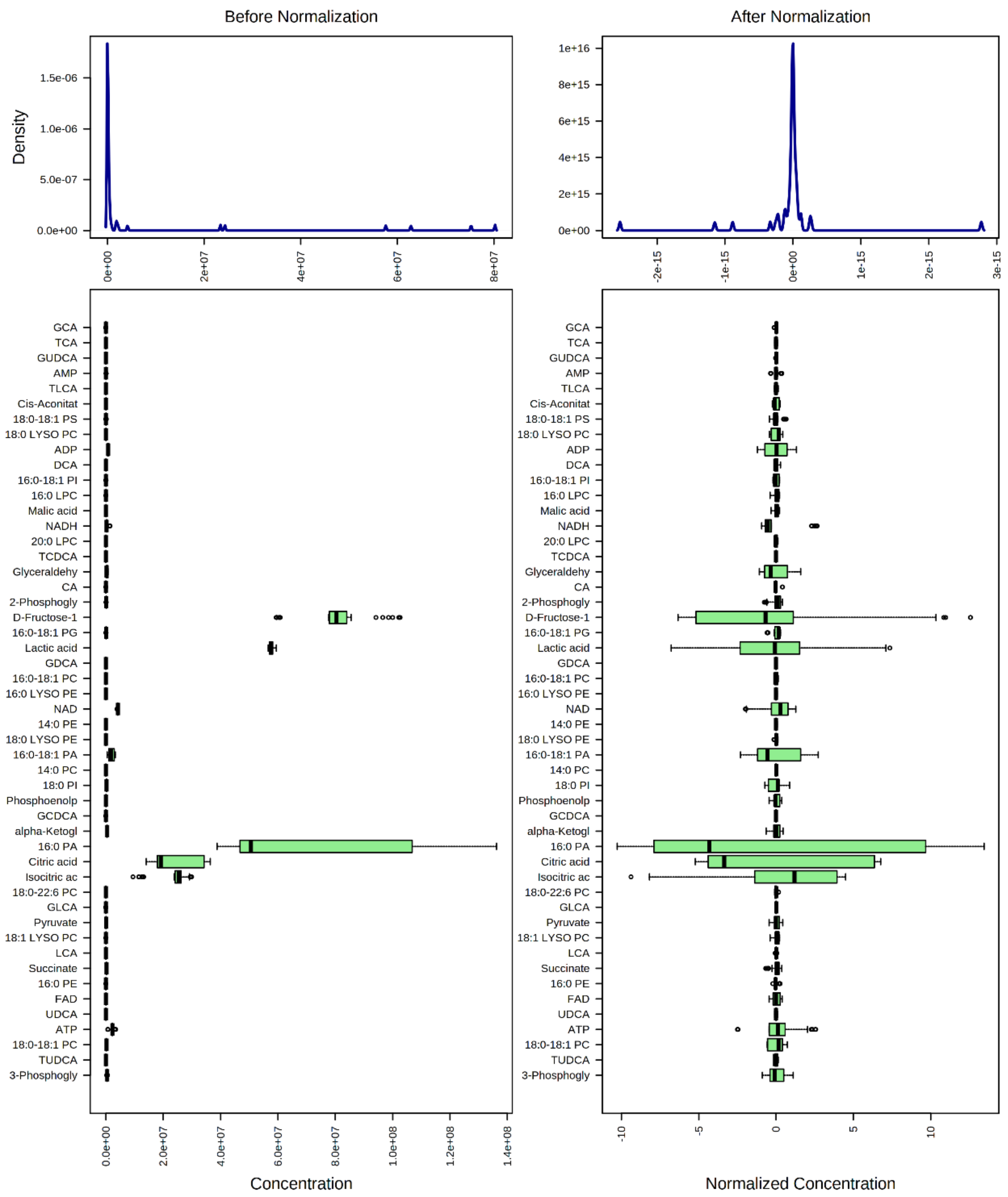

Figure S4. Data of targeted metabolism were compared before and after normalization.

Table S1. Target compound information.

No.	Analytes	Formula	CAS No.	Common Name
1	Pyruvate	C3H4O3	127-17-3	Pyruvic acid
2	Lactic acid	C3H6O3	50-21-5	2-Hydroxypropanoic acid
3	Fumarate	C4H4O4	110-17-8	Fumaric acid
4	Oxaloacetic acid	C4H4O5	328-42-7	Oxaloacetate
5	Succinate	C4H6O4	110-15-6	Amber Acid
6	Malic acid	C4H6O5	617-48-1	Malic acid
7	α-Ketoglutaric acid	C5H6O5	328-50-7	α-Ketoglutaric acid
8	Phosphoenolpyruvate	C3H5O6P	9067-77-0	Phosphoenolpyruvic acid
9	Glyceraldehyde 3-phosphate	C3H7O6P	142-10-9	Glyceraldehyde 3-phosphate
10	Cis-Aconitate	C6H6O6	585-84-2	Cis-Aconitate
11	Glucose	C6H12O6	50-99-7	D-Glucose
12	2-Phosphoglyceric acid	C3H7O7P	2553-59-5	DL-2-phosphoglyerate

13	3-Phosphoglyceric acid	C3H7O7P	820-11-1	DL-3-phosphoglyerate
14	Citric acid	C6H8O7	77-92-9	Citric acid
15	Isocitric acid	C6H8O7	320-77-4	Isocitric acid
16	Glucose 6-Phosphate	C6H12O6·PO3H	56-73-5	Glucose 6-Phosphate
17	D-Fructose-1,6-Diphosphate	C6H11Na3O12P2	38099-82-0	D-Fructose 1,6-Bisphosphate Trisodium Salt
18	AMP	C10H14N5O7P	61-19-8	Adenosine monophosphate
19	ADP	C10H15N5O10P2	58-64-0	5'-adenylphosphoric acid
20	ATP	C10H16N5O13P3	9000-83-3	Adenosine 5'-triphosphatase
21	NAD	C21H27N7O14P2	53-84-9	β-Nicotinamide adenine dinucleotide
22	NADH	C21H30N7NaO14P2	606-68-8	beta-Nicotinamide adenine dinucleotide disodium salt
23	FAD	C27H33N9Na2O16P2	84366-81-4	Flavin adenine dinucleotide disodium salt hydrate
24	Acetyl CoA	C23H38N7O17P3S.3Na	102029-73-2	Acetyl CoA
25	TCA	C26H45NO7S	81-24-3	Taurocholic acid
26	GCA	C26H43NO6	475-31-0	Glycocholic acid
27	CA	C24H40O5	81-25-4	Cholic acid
28	TCDCA	C26H45NO6S	516-35-8	Taurochenodeoxycholic Acid
29	GCDCA	C26H43NO5	64480-66-6	Chenodeoxycholic acid glycine conju-gate
30	TDCA	C26H45NO6S	207737-97-1	Taurodeoxycholic acid
31	CDCA	C24H40O4	474-25-9	Chenodeoxycholic acid
32	GDCA	C26H43NO5	360-65-6	Deoxycholic acid glycine conjugate
33	DCA	C24H40O4	83-44-3	Desoxycholic acid
34	TLCA	C26H45NO5S	6042-32-6	Taurolithocholic acid
35	GLCA	C26H43NO4	474-74-8	Lithocholic acid glycine conjugate
36	LCA	C24H40O3	434-13-9	Lithocholic acid
37	TUDCA	C26H45NO6S	14605-22-2	Tauroursodeoxycholic acid
38	GUDCA	C26H43NO5	64480-66-6	Glycylursodeoxycholic acid
39	UDCA	C24H40O3	128-13-2	Ursodeoxycholic acid
40	14:0 PC	C36H72NO8P	18194-24-6	1,2-dimyristoyl-sn-glycero-3-phospho-choline
41	16:0 PC	C40H80NO8P	63-89-8	1,2-dipalmitoyl-sn-glycero-3-phospho-choline
42	16:0-18:0 PC	C42H84NO8P	59403-51-9	1-palmitoyl-2-stearoyl-sn-glycero-3-phosphocholine
43	16:0-18:1 PC	C42H82NO8P	26853-31-6	1-palmitoyl-2-oleoyl-sn-glycero-3-phos-phocholine
44	16:0-22:4 PC	C44H80NO8P	35418-58-7	1-palmitoyl-2-arachidonoyl-sn-glycero-3-phosphocholine
45	18:0 PC	C44H88NO8P	816-94-4	1,2-distearoyl-sn-glycero-3-phosphocho-line
46	18:0-18:1 PC	C44H86NO8P	56421-10-4	1-stearoyl-2-oleoyl-sn-glycero-3-phos-phocholine
47	18:0-22:6 PC	C48H84NO8P	59403-52-0	1-stearoyl-2-docosahexaenoyl-sn-glyc-ero-3-phosphocholine
48	20:0 PC	C48H96NO8P	61596-53-0	1,2-diarachidoyl-sn-glycero-3-phospho-choline
49	16:0 Lyso PC	C24H50NO7P	17364-16-8	1-palmitoyl-2-hydroxy-sn-glycero-3-phosphocholine
50	18:1 Lyso PC	C26H52NO7P	19420-56-5	1-oleoyl-2-hydroxy-sn-glycero-3-phos-phocholine
51	18:0 Lyso PC	C26H54NO7P	19420-57-6	1-stearoyl-2-hydroxy-sn-glycero-3-phos-phocholine
52	20:0 Lyso PC	C28H58NO7P	108341-80-6	1-heptadecanoyl-2-hydroxy-sn-glycero-3-phosphocholine
53	16:0 PA	C35H68NaO8P	169051-60-9	1,2-dipalmitoyl-sn-glycero-3-phosphate (sodium salt)
54	16:0-18:1 PA	C37H70O8PNa	169437-35-8	1-palmitoyl-2-oleoyl-sn-glycero-3-phos-phate (sodium salt)

55	16:0 Lyso PA	C19H38O7PNa	17618-08-5	1-palmitoyl-2-hydroxy-sn-glycero-3-phosphate (sodium salt)
56	16:0 PS	C38H73NO10PNa	145849-32-7	1,2-dipalmitoyl-sn-glycero-3-phospho-L-serine (sodium salt)
57	18:0-18:1 PS	C42H79NO10PNa	321883-23-2	1-stearoyl-2-oleoyl-sn-glycero-3-phospho-L-serine (sodium salt)
58	18:0 Lyso PS	C24H47NPO9Na	1246298-16-7	1-stearoyl-2-hydroxy-sn-glycero-3-phospho-L-serine
59	16:0 PI	C41H82NO13P	34290-57-8	1,2-dipalmitoyl-sn-glycero-3-phospho-(1'-myo-inositol)
60	18:0 PI	C45H90NO13P	849412-67-5	1,2-distearoyl-sn-glycero-3-phosphoinositol
61	16:0-18:1 PI	C43H84NO13P	50730-13-7	1-palmitoyl-2-oleoyl-sn-glycero-3-phosphoinositol
62	18:0-20:4 PI	C47H86NO13P	1331751-28-0	1-stearoyl-2-arachidonoyl-sn-glycero-3-phosphoinositol
63	18:0 Lyso PI	C27H56NO12P	849412-49-3	1-stearoyl-2-hydroxy-sn-glycero-3-phosphoinositol
64	14:0 PG	C34H66O10PNa	200880-40-6	1,2-dimyristoyl-sn-glycero-3-phospho-(1'-rac-glycerol)
65	16:0-18:1 PG	C40H76O10PNa	268550-95-4	1-palmitoyl-2-oleoyl-sn-glycero-3-phospho-(1'-rac-glycerol)
66	18:0 PG	C42H82O10PNa	200880-42-8	1,2-distearoyl-sn-glycero-3-phospho-(1'-rac-glycerol)
67	14:0 PE	C33H66NO8P	998-07-2	1,2-dimyristoyl-sn-glycero-3-phosphoethanolamine
68	16:0 PE	C37H74NO8P	923-61-5	1,2-dipalmitoyl-sn-glycero-3-phosphoethanolamine
69	18:0 PE	C41H82NO8P	1069-79-0	1,2-distearoyl-sn-glycero-3-phosphoethanolamine
70	16:0 Lyso PE	C21H44NO7P	53862-35-4	1-palmitoyl-2-hydroxy-sn-glycero-3-phosphoethanolamine
71	18:0 Lyso PE	C23H48NO7P	69747-55-3	1-Stearoyl-2-Hydroxy-sn-Glycero-3-Phosphoethanolamine
72	d_4-Succinic acid	C4H2D4O4	14493-42-6	d_4-Succinic acid
73	^{13}C-AMP	^{13}C5C510H14N5O7P	-	[13C5]-Adenosine 5’-Monophosphate
74	^{13}C-GCA	C25H43NO6C13	-	Glycocholic acid-C13
75	17:0 Lyso PC	C25H52NO7P	50930-23-9	1-heptadecanoyl-2-hydroxy-sn-glycero-3-phosphocholine
76	17:0 Lyso PA	C20H40O7PNa	799268-66-9	1-heptadecanoyl-2-hydroxy-sn-glycero-3-phosphate
77	17:1 Lyso PS	C23H43NNaO9P	1246298-15-6	1-(10Z-heptadecenoyl)-2-hydroxy-sn-glycero-3-[phospho-L-serine]
78	17:1 Lyso PI	C26H52NO12P	1246353-39-8	1-(10Z-heptadecenoyl)-2-hydroxy-sn-glycero-3-phospho-(1'-myo-inositol)
79	15:0 PG	C36H70O10PNa	322647-32-5	1,2-dipentadecanoyl-sn-glycero-3-phospho-(1'-rac-glycerol)
80	17:1 Lyso PE	C22H44NO7P	1246298-09-8	1-(10Z-heptadecenoyl)-sn-glycero-3-phosphoethanolamine

Table S2. Mass spectrum parameters of target compounds.

Compound	Q1 Mass(Da)	Q3 Mass(Da)	DP(volts)	CE(volts)
Malic acid	133.0	115.0	-40	-15
α-Ketoglutaric acid	145.1	1010	-15	-15
Citric acid	191.0	111.0	-15	-20
Oxaloacetic acid	131.1	87.0	-60	-15
Lactic acid	89.1	89.0	-60	-10
Fumarate	115.0	71.0	-50	-10
Phosphoenolpyruvate	167.0	79.0	-10	-20
Succinate	117.0	73.0	-40	-20
Pyruvate	87.0	43.0	-30	-20
Cis-Aconitate	173.0	85.0	-25	-20
Glucose	179.1	89.0	-60	-10
3-Phosphoglyceric acid	185.1	79.0	-40	-50
Glucose 6-Phosphate	275.0	79.0	-35	-75
Glyceraldehyde 3-phosphate	169.0	97.0	-15	-15
D-Fructose-1,6-Diphosphate	339.0	79.0	-35	-80
Isocitric acid	191.1	111.0	-20	-20
2-Phosphoglyceric acid	185.0	79.0	-50	-55
Acetyl CoA	808.1	808.1	-40	-10
NAD	662.1	540.0	-50	-30
ATP	506.0	159.0	-70	-60
ADP	426.0	79.0	-70	-100
AMP	346.0	79.0	-80	-100
NADH	664.1	664.0	-120	-10
FAD	784.1	784.0	-100	-10
Glucose	179.1	59.0	-60	-25
Oxaloacetic acid	131.1	131.1	-60	-10
TCA	514.4	514.4	-146	-30
GCA	464.4	464.4	-160	-27
CA	407.2	407.2	-132	-27
CA	407.2	289.2	-132	-53
TCDCA	498.4	498.4	-187	-30
GCDCA	448.4	448.4	-63	-28
TDCA	498.0	402.0	-130	-34
CDCA	391.2	391.2	-153	-30
GDCA	448.0	402.0	-140	-12
DCA	391.0	201.0	-150	-12
TLCA	482.4	482.4	-150	-30
GLCA	432.2	432.2	-145	-25
LCA	375.2	375.2	-150	-17
TUDCA	498.0	401.0	-150	-11
GUDCA	448.0	401.0	-55	-30
UDCA	391.0	202.0	-130	-12
14:0 PC	722.6	227.2	-75	-50
16:0 PC	778.5	718.7	-75	-30
16:0-18:0 PC	806.5	255.3	-75	-50
16:0-18:1 PC	804.6	480.0	-75	-30
16:0-22:4 PC	826.6	227.0	-143	-37
18:0 PC	834.6	283.4	-75	-50
18:0-18:1 PC	832.6	283.4	-75	-50
18:0-22:6 PC	878.6	283.4	-75	-50
20:0 PC	890.6	311.4	-75	-50
16:0 Lyso PC	540.3	224.0	-95	-38
18:1 Lyso PC	566.4	468.5	-95	-31
18:0 Lyso PC	568.4	224.4	-86	-42
16:0 Lyso PE	453	255.3	-75	-25
18:0 Lyso PE	480.3	283.4	-75	-25
20:0 Lyso PC	552.3	104.1	120	30
16:0 PA	680.6	663.5	135	13
16:0-18:1 PA	675.6	338.3	130	24

16:0 Lyso PA	432.9	313.5	75	32
16:0 PS	758.6	208.0	75	35
18:0-18:1 PS	812.6	208.0	75	35
18:0 Lyso PS	548.4	208.1	75	35
16:0 PI	828.6	551.6	75	23
18:0 PI	884.6	607.6	75	23
16:0-18:1 PI	854.6	577.6	75	23
18:0-20:4 PI	904.6	627.6	75	23
18:0 Lyso PI	618.4	341.4	75	30
14:0 PG	689.2	195.0	75	34
16:0-18:1 PG	771.6	195.0	75	34
18:0 PG	801.7	194.9	75	34
14:0 PE	636.5	495.5	75	28
16:0 PE	692.5	551.5	75	28
18:0 PE	768.6	376.4	89	49
d_4-Succinate	121.0	77.0	-25	-20
^{13}C AMP	351.2	79.0	-85	-60
d-GCA	465.4	465.4	-157	-11
17:0 Lyso PC	554.4	269.3	-75	-40
17:1 Lyso PE	464.3	267.4	-75	-25
17:0 Lyso PA	447.2	327.4	75	32
17:1 Lyso PS	532.3	208.1	75	35
17:1 Lyso PI	602.4	325.4	75	30
15:0 PG	717.6	194.9	75	34

Table S3. Design of Primer Sequences.

Target gene	Forward(5'to 3')	Reverse(5'to 3')
LCAT	TGGCTCCTCAATGTGCTCTTCC	CACCACATCTGGTTTGTCCAGC
GAPDH	AGATCCCTCCAAAATCAAGTGG	GGCAGAGATGATGACCCTTTT
